# Supplementary material for: Genome-Wide Analysis of the Growth-Regulating Factor Family in Peanut (Arachis hypogaea L.)
Source: Int J Mol Sci. 2019 Aug 23;20(17):4120. doi: 10.3390/ijms20174120 (PMC6747334; doi:10.3390/ijms20174120)
Supplement: Supplementary file 1 [file ijms-20-04120-s001.zip › ijms-573253 supplementary figures.pdf]

Supplementary materials

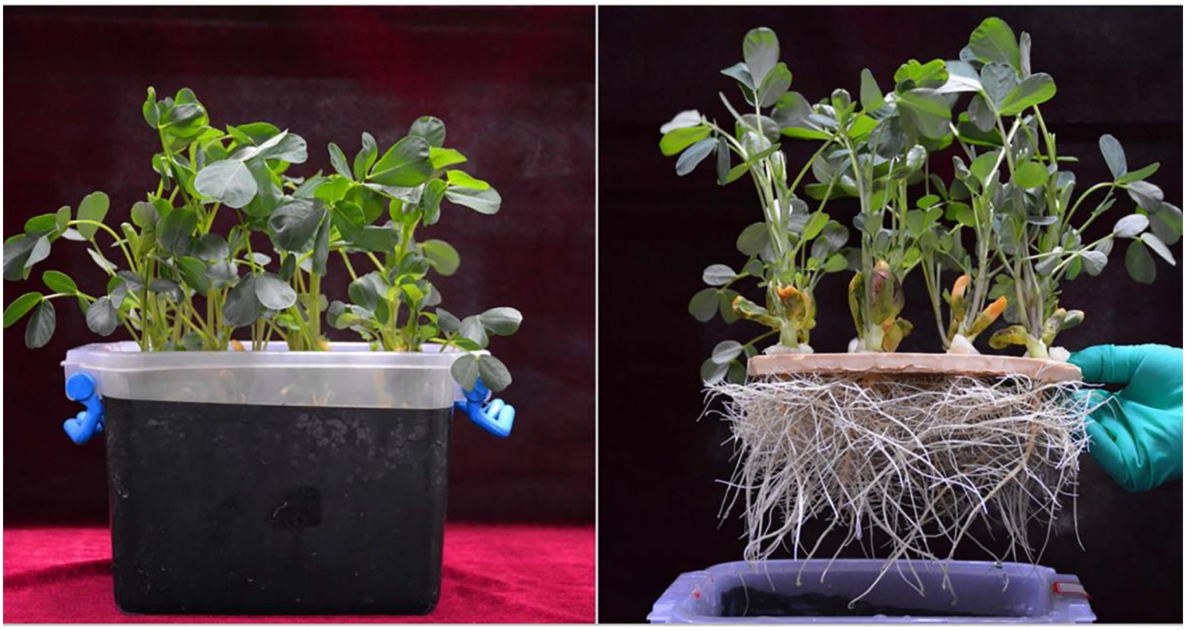

Figure S1. Two-week-old seedlings of H8107 grown on plates cultivated with 1/2 Hoagland solution.

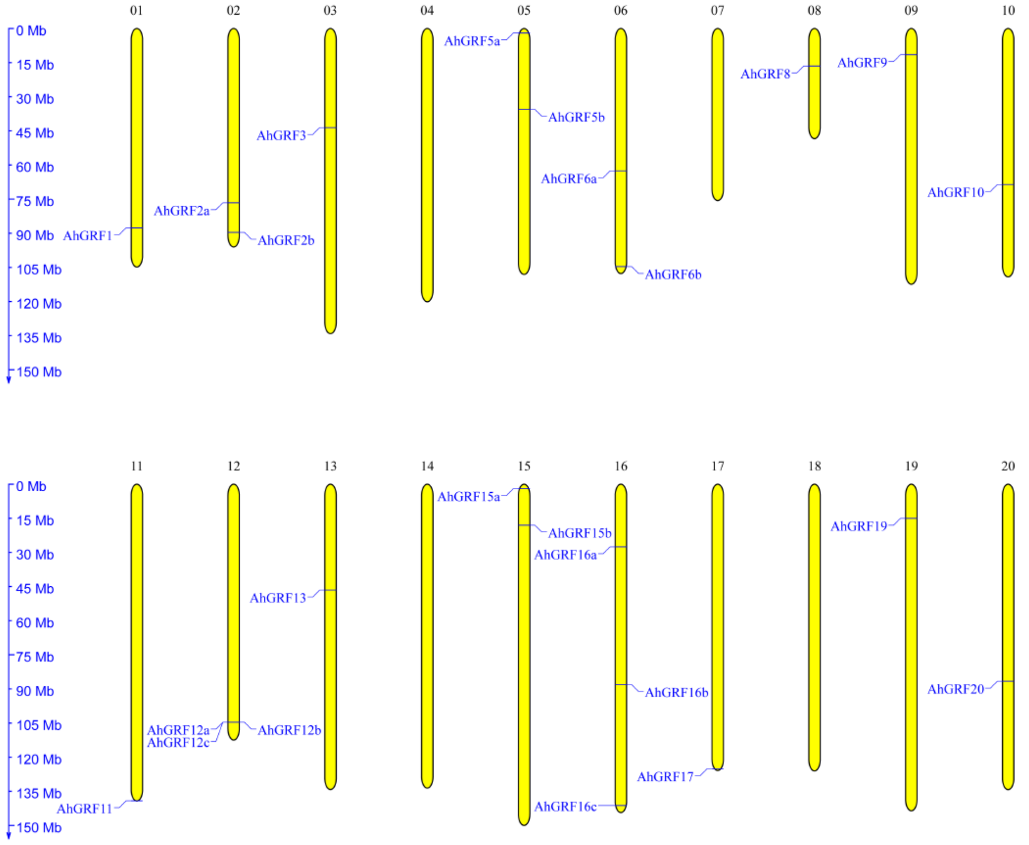

Figure S2. Distribution of *AhGRF* genes in the cultivated peanut genome. The chromosomal position of each *AhGRF* genes was mapped to the cultivated peanut genome. The chromosome number is indicated above each chromosome.

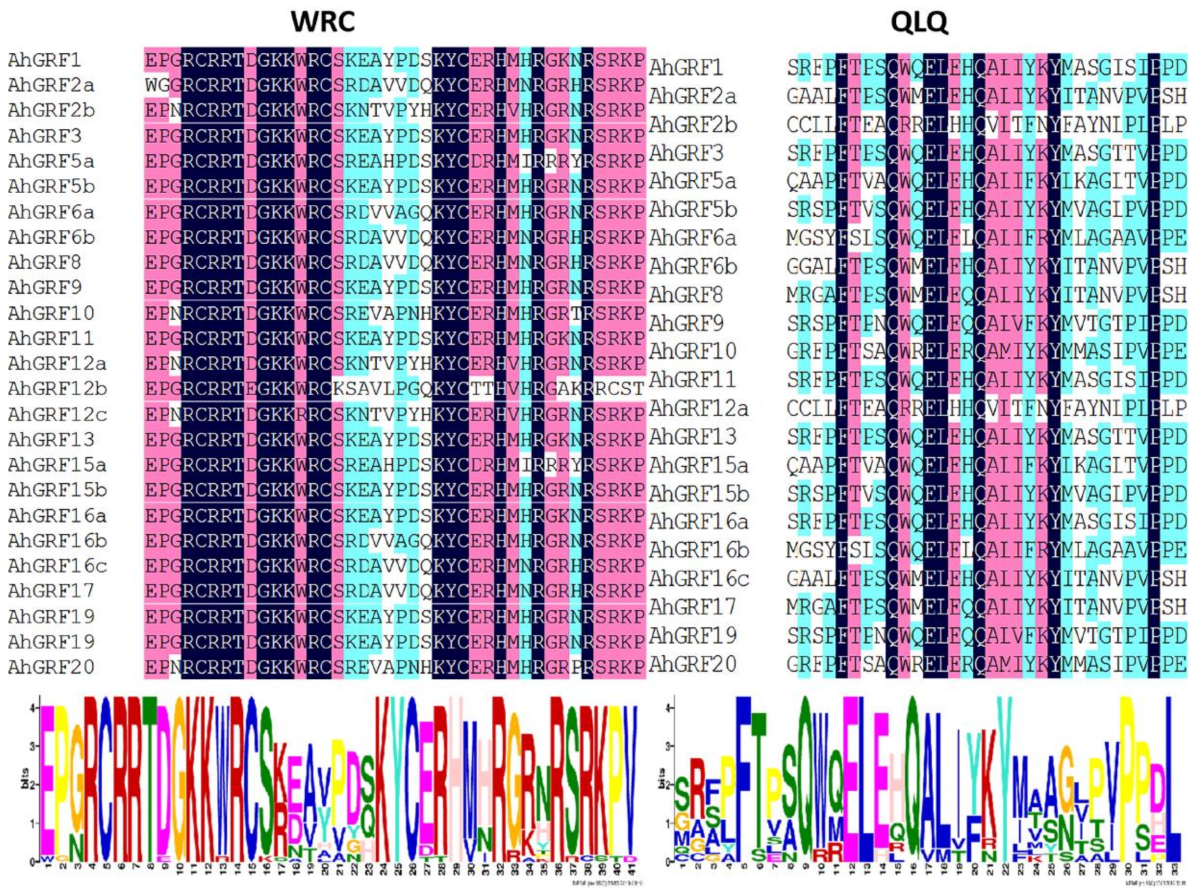

Figure S3. Sequence alignment of WRC and QLQ motifs in *AhGRF* genes.
